# An observational study of headaches in children and adolescents with functional abdominal pain: Relationship to mucosal inflammation and gastrointestinal and somatic symptoms

**DOI:** 10.1097/MD.0000000000011395

**Published:** 2018-07-27

**Authors:** Craig Friesen, Meenal Singh, Vivekanand Singh, Jennifer V. Schurman

**Affiliations:** a Division of Gastroenterology, Hepatology, and Nutrition; b Department of Pathology and Laboratory Medicine; c Division of Developmental and Behavioral Sciences, Children's Mercy Kansas City, Kansas City, MO.

**Keywords:** abdominal pain, functional dyspepsia, headache, irritable bowel syndrome, mast cells

## Abstract

Headaches and abdominal pain are among the most common pediatric pain conditions. Mast cells have been implicated in the pathophysiology of migraines, as well as functional dyspepsia (FD) and irritable bowel syndrome (IBS). The primary aims of the current study were to assess headache prevalence in patients with FD and to assess the association between headaches and mucosal mast cells and eosinophils. An additional aim was to explore associations of headache with other symptoms.

We conducted a cross-sectional retrospective chart review of 235 consecutive patients with chronic abdominal pain. All patients had completed a standardized questionnaire as part of their routine clinical evaluation. Both gastrointestinal and non-gastrointestinal somatic symptoms were included in the analysis. All patients diagnosed with FD had undergone upper endoscopy with biopsies obtained from the gastric antrum and duodenum and these specimens were utilized to assess eosinophil and mast cell densities, respectively.

Overall, 86% of patients fulfilled Rome IV criteria for FD. Headache was reported by 73.8% of FD patients versus 45.2% of non-FD patients (*P* = .001). Duodenal mast cell densities were significantly increased in those reporting headaches. Headache was not associated with any specific gastrointestinal symptoms but was associated with a wide array of non-gastrointestinal symptoms including fatigue, dizziness, muscle pain, joint pain, and chest pain.

Headaches are common in children and adolescents with abdominal pain and, utilizing Rome IV criteria, are specifically associated with FD. In patients with FD, headaches are associated with increased duodenal mast cell density and a variety of somatic symptoms, all of which are possibly the result of mast cell activation.

## 1 Introduction

Pediatric chronic pain is increasingly recognized as a significant public health issue, with headaches and abdominal pain among the most commonly reported pain conditions.^[1]^ Prevalence rates for headaches in children and adolescents currently is estimated at over 50%, while systematic reviews report specific migraine headache prevalence of approximately 8% to 9%.^[2,3]^ Prevalence rates for both migraine and non-migraine headache types appear to be increasing significantly over time.^[4]^ Chronic or recurrent abdominal pain is estimated to affect up to 19% of the pediatric population, with the majority reporting symptoms that fit into discrete diagnostic categories under the broader heading of functional gastrointestinal disorders (FGIDs).^[5,6]^ Understanding associations between these 2 common pediatric pain conditions may help to better define the pathophysiology underlying both.

Similar to pediatric headache, criteria exist to subdivide the population of youth with abdominal pain into discrete categories. These criteria, commonly referred to as the Rome Criteria, were initially defined and subsequently revised by expert panels. Currently, there are 4 FGIDs related to abdominal pain with the 2 most common being functional dyspepsia (FD) and irritable bowel syndrome (IBS).^[7,8]^ The most current version of the criteria, Rome IV, was released in 2016 and significantly altered the criteria for FD and IBS. Previously, under Rome III, FD was defined as upper abdominal pain or discomfort unrelated to stools.^[6]^ Under Rome IV, FD is defined by the presence of epigastric abdominal pain unrelated to stools, early satiety, or postprandial fullness.^[9]^ IBS is defined by the presence of pain related to a change in stool frequency, a change in stool consistency, or by a change in pain with stooling. Previously, Rome III criteria required two of these symptoms to be present while Rome IV criteria only requires one of these symptoms to be present.^[6,9]^

Some literature exists to suggest that abdominal pain symptoms may be more common in the headache population, and vice versa. Previous studies of adults with headache have demonstrated an increased incidence of upper abdominal symptoms, while migraine headaches in youth have been associated with both FD and IBS, as defined by Rome III.^[10,11]^ Le Gal et al^[11]^ reported an increase of FD, IBS, and abdominal migraines (another FGID), respectively, in children and adolescents presenting to an emergency department with migraine headaches, as compared to pediatric patients presenting with mild trauma and no history of headaches. Similarly, headache and other somatic complaints appear common in pediatric patients with FGIDs. Specifically, migraine-like headaches have previously been reported in 40% of children with FGIDs.^[12]^

The association between headaches and chronic abdominal pain opens up the possibility of common pathophysiologic triggers or mechanisms. There are several contributors that these 2 pain conditions share. Stress and anxiety have been highly implicated in both conditions and are generally viewed as frequent significant contributors.^[13–16]^ To a lesser degree, environmental allergens have been implicated in both headaches and FD.^[17,18]^ Taken together, these contributors would seem to suggest a common process involving mast cells.

To date, mast cells have been implicated in the pathophysiology of migraines, as well as FD and IBS.^[19–21]^ In a sample of 56 youth with FD (as defined by Rome III), Yeom et al^[22]^ reported an increase in gastric body and duodenal mast cells for patients who also reported headaches, as compared to those without headaches. This association appeared specific, as they found no associations with lymphocytes, neutrophils, and enteroendocrine cells based on headache status. Eosinophils, which share many physiologic processes with mast cells, were not evaluated. In adults with IBS, mast cells have been extensively evaluated. Generally, results have demonstrated increased mast cell density and degranulation; further, the density of mast cells in close proximity to nerves correlates with pain frequency and intensity.^[20,23,24]^ These associations make intuitive sense, as mast cells may be activated by a number of factors which have been implicated in both headaches and abdominal pain, including stress and allergen exposure.^[25]^

The primary aims of the current study were to assess headache prevalence in patient with FD and IBS, respectively, and to assess the association between headaches and densities of gastric and duodenal mast cells and eosinophils, respectively, in children with FD defined by Rome IV. This second aim was limited to FD patients because, unlike IBS, mucosal biopsy is a standard part of the diagnostic evaluation of dyspepsia yielding an unselected clinical group for comparison. An additional aim was to explore associations of headache with specific gastrointestinal and somatic symptoms.

## 2 Methods

We conducted a cross-sectional retrospective chart review of a convenience sample of 235 consecutive patients diagnosed with an FGID by a single board-certified pediatric gastroenterologist in a subspecialty abdominal pain clinic at Children's Mercy Kansas City. This study was approved by the Institutional Review Board of Children's Mercy Kansas City. Patients ranged in age from 8 to 17 years and reported having abdominal pain at least once weekly for a minimum of 8 weeks. All patients had completed a standardized questionnaire as part of their routine clinical evaluation. The questionnaire asked about gastrointestinal symptoms used to classify symptoms according to Rome IV criteria, as well as an array of non-gastrointestinal symptoms. Both gastrointestinal and non-gastrointestinal somatic symptoms were included in the analysis.

All patients diagnosed with FD had undergone upper endoscopy with biopsies obtained from the esophagus, gastric antrum, and duodenum after failing to respond to acid reduction therapy. All patients were negative for nodularity, erosions, ulcers, and *Helicobacter pylori*. Their previously obtained biopsy specimens were utilized to assess eosinophil and mast cell densities, respectively, in both the gastric antrum and the duodenum. Hematoxylin and eosin (H & E) stained slides, obtained from these patients as part of routine care, were used to assess eosinophil density. An immunohistochemical stain for tryptase was performed on the antral and duodenal biopsies of all study patients to determine mast cell densities. A mouse monoclonal antibody, anti-human mast cell tryptase, from Dako (clone AA1) was used at a dilution of 1:2000 on the Bond automated immunostainer following routine immunohistochemistry protocol for the stainer.

To determine eosinophil and mast cell densities, sections were initially scanned at a low magnification (× 10 objective magnification) to determine areas of maximal density. Using an Olympus CH30 microscope with a combination of 40 × objective and 10 × eyepiece, cells were counted in 5 consecutive high-power fields. Both cell types were counted only in the lamina propria of the mucosa. For the eosinophils, only cells demonstrating its nucleus were enumerated. For the mast cells, both spindled and epithelioid forms showing a nucleus were enumerated. Peak and mean cell densities were determined for the eosinophils and mast cells, respectively, in both the antrum and the duodenum. All cell counts were performed by a single observer (MS).

## 3 Statistical analysis

SPSS version 23 (SPSS Inc., Chicago, IL) was used to perform statistical analysis. The frequency of a positive report of headache was compared between patients who met criteria for FD and IBS, respectively, as compared to those who did not meet criteria for each variable independently. All subsequent analyses were performed only on patients meeting FD criteria. Mean and peak eosinophil and mast cell densities, respectively, were compared between patients reporting headaches and those who did not by the student *t* test. Frequencies of specific gastrointestinal and non-gastrointestinal somatic symptoms were compared between patients reporting headaches and those who did not by chi square analysis. A *P* < .05 was considered statistically significant for all analyses.

## 4 Results

Overall, 86% of patients fulfilled Rome IV criteria for FD and 58% fulfilled criteria for IBS. Fifty percent of patients fulfilled criteria for both FD and IBS. Overall, headache was reported by 69.8% of the patients. Headache was reported by 73.8% of FD patients (regardless of IBS status) versus 45.2% of non-FD patients (*P* = .001). Headache was reported by 57.9% of IBS (regardless of FD status) patients versus 56.3% of non-IBS patients (*P* = .82). As headache was only associated with FD, all other analyses were performed only with FD patients.

For the FD patients, 77.9% were female and the mean age was 13.2 ± 2.6 years. The duration of abdominal pain ranged from 2 to 190 months with a mean of 30.0 ± 38.0 months. Abdominal pain occurred daily in 75.5%, several times per week in 17.2%, and weekly in 7.3%. Early satiety was reported by 80.4%, postprandial bloating by 58.8%, and epigastric pain by 48.0% of patients. Eosinophil and mast cell densities by location for FD patients with and without headache are shown in Table 1. Duodenal mean (20.5 ± 7.7 vs 17.4 ± 7.8; *P* = .022) and peak (26.5 ± 9.6 vs 23.0 ± 10.0; *P* = .047) mast cell densities were significantly increased in those reporting headaches.

**Table 1:**
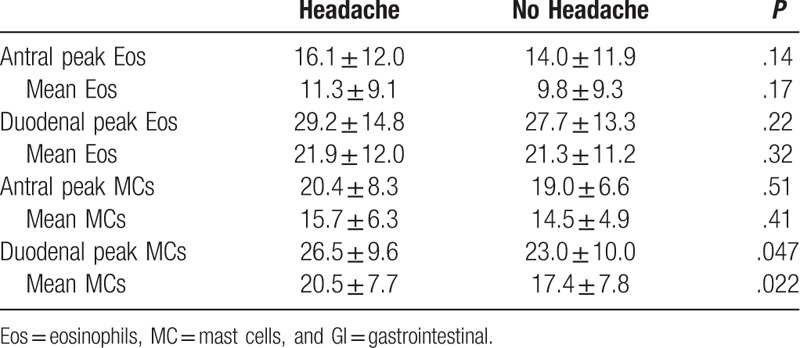
Eosinophil and mast cell densities in patients with headache versus no headache.

The frequencies of gastrointestinal and non-gastrointestinal symptoms for patients with and without headache are shown in Table 2. There were no significant associations between headache and specific gastrointestinal symptoms in isolation. However, headache was associated with a wide array of specific non-gastrointestinal somatic symptoms.

**Table 2:**
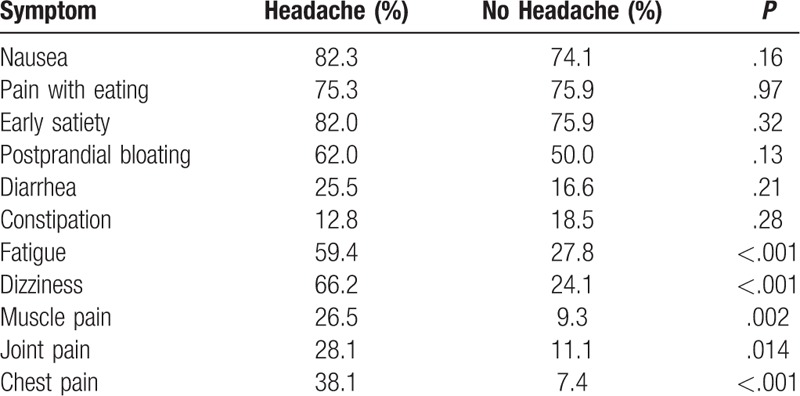
Frequencies of GI and non-GI Symptoms in patients with headache versus no headache.

## 5 Discussion

Headaches are common in patients with pain-associated FGIDs being reported by nearly 70% of patients in the current study. In the current study evaluating consecutive patients referred to an abdominal pain clinic, we found headaches to be specifically and uniquely associated with a diagnosis of FD, by Rome IV criteria, when compared to abdominal pain patients not meeting criteria for FD. Differences from previous studies may be accounted for, in part, by the differences in FD criteria between Rome III, which was used in previous studies, as compared to Rome IV, which was utilized in the current study. Specifically, the criteria for FD became narrower, while the criteria for IBS were liberalized, creating a more heterogeneous group of patients who would be classified as IBS, or non-FD more generally.

In the current study, the presence of headaches was associated with increased duodenal mast cell density in patients with FD as defined by Rome IV criteria. This is consistent with the findings of Yeom et al,^[22]^ who demonstrated increased mast cell density in the body (but not the antrum) of the stomach and the duodenum of patients fulfilling Rome III criteria for FD who were also experiencing headaches. They found no association with other inflammatory or enteroendocrine cells, just as we found no association with eosinophils; this suggests that the immune response as related to headaches may be limited to, or primarily driven by, mast cells. Mast cell biologic effects are the result of mediator release, with subsequent mediator-driven actions. Thus, biologic effects are not only a function of mast cell density, but also the degree of mast cell mediator production and release. We have previously demonstrated active degranulation of mucosal mast cells in children with FD, providing initial support for this pathway.^[26]^

The potential mechanism of mast cell involvement in the generation of headache and FD may be demonstrated more clearly by examining the stress response, as anxiety and stress have been highly implicated in both pain conditions. A main feature of the stress response is the release of corticotropin releasing hormone (CRH). Mast cells express CRH receptors which, when activated, result in the release of inflammatory cytokines. Once released, mast cell mediators, including histamine and leukotrienes, can stimulate afferent nerves signaling pain or can sensitize afferent nerves resulting in hypersensitivity and changes in electromechanical function. In children and adolescents, mucosal mast cell density correlates with higher anxiety scores, slower gastric emptying, and gastric electrical dysrhythmias.^[26,27]^ Adults have demonstrated luminal release of tryptase and histamine from jejunal mast cells under stress conditions at a magnitude similar to that seen in allergic food reactions.^[28]^

Histamines, as well as leukotrienes, have been implicated in both headaches and FD. Histamine infusion induces headaches in patients with muscle contraction and migraine headaches, but not in controls without headache; further, this effect is blocked by H1 antagonism and, to a lesser degree, by H2 antagonism.^[29,30]^ Combining an H1 antagonist with an H2 antagonist has been reported to relieve pain in 50% of children with FD associated with mucosal eosinophilia and in 79% of adults with FD associated with increased mucosal mast cell density who had previously failed to respond to acid reducing medications.^[31,32]^ Increased urinary leukotrienes (LT-E4) has been shown to increase significantly during migraine headaches in children.^[33]^ Although there are not controlled trials, an open-label trial of a leukotriene inhibitor, montelukast, demonstrated a > 50% reduction in migraine headache occurrence in over half of a group of adults with migraine headaches.^[34]^ Montelukast also has demonstrated efficacy for relief of pain in a controlled trial in children and adolescents with FD.^[35]^ The use of montelukast to treat migraine headaches or FD should be considered investigational.

In the current study, headaches were associated with a Rome IV diagnosis of FD, but not any specific gastrointestinal symptoms in isolation. In contrast, patients with headaches reported fatigue, dizziness, muscle pain, joint pain, and chest pain more frequently than did patients without headaches. Previous community studies have demonstrated that abdominal pain in children and adolescents is associated with high rates of somatic symptoms, especially in those who fulfill Rome criteria for an FGID.^[36,37]^ Our previous studies have demonstrated an association between mucosal mast cell density and somatic complaints and, further, that headaches cluster with the same somatic symptoms as found in the current study.^[27,38]^ Consistent with the current study, non-gastrointestinal somatic symptoms were uniquely associated with the presence of an FD diagnosis in the previous study.^[38]^ Of note, all of these non-gastrointestinal somatic symptoms have previously been reported to occur with mast cell activation.^[39]^

Strength of the current study is the systematic collection of symptoms in a large population of children and adolescents. A weakness is that because the data was collected in an abdominal pain clinic, the specific headache type is not routinely determined and therefore could not be analyzed. Future studies should evaluate mucosal mast cells in relation to the specific type of headache.

In conclusion, headaches are common in children and adolescents with abdominal pain and, utilizing Rome IV criteria, are specifically associated with FD. In patients with FD, headaches are associated with increased duodenal mast cell density and a variety of somatic symptoms, all of which are possibly the result of mast cell activation. Future studies should evaluate headache treatment responses to mast cell stabilizers or antagonists of specific mast cell mediators in children and adolescents with FD.

## Author contributions

**Conceptualization:** Craig Friesen, Meenal Singh, Vivekanand Singh, Jennifer Schurman.

**Data curation:** Craig Friesen, Jennifer Schurman.

**Formal analysis:** Craig Friesen, Meenal Singh, Vivekanand Singh, Jennifer Schurman.

**Investigation:** Craig Friesen, Meenal Singh, Vivekanand Singh.

**Methodology:** Craig Friesen, Meenal Singh, Vivekanand Singh, Jennifer Schurman.

**Project administration:** Craig Friesen.

**Writing – original draft:** Craig Friesen.

**Writing – review & editing:** Craig Friesen, Meenal Singh, Vivekanand Singh, Jennifer Schurman.
